# 

**Published:** 1886-01

**Authors:** 


### COLLABORATORS.

### CHICAGO:

PROFESSOR J. A. ALLEN, M. D., LL. D.

PROFESSOR E. ANDREWS, M. D., LL. D.

PROFESSOR W. H. BYFORD, A. M., M. D.

PROFESSOR O. C. DEWOLF, A. M., M. D.

PROFESSOR E. C. DUDLEY, A. B., M. D.

PROFESSOR J. H. ETHERIDGE, A. M., M. D.

PROFESSOR C. FENGER, M. D.

PROFESSOR J. N. HYDE, A. M., M. D

PROFESSOR E. F. INGALS, A. M., M.D.

PROFESSOR H. A. JOHNSON, M. D., LL. D.

CHARLES GILMAN SMITH, A. M., M.D,

J. F. TODD, M. D.

### MILWAUKEE, WISCONSIN:

PROFESSOR N. SENN, M. D.

### WASHINGTON, D. C:

S. O. RICHEY, M. D.

### UNITED STATES ARMY:

SURGEON, D. L. HUNTINGTON, M. D.

### UNITED STATES NAVY:

MEDICAL DIRECTOR, J. M. BROWNE, M. D

MEDICAL DIRECTOR, A. L. GIHON, A. M., M. D.

---

### CONTRIBUTORS OF ORIGINAL ARTICLES

J. H. ETHERIDGE,
E. J. DOERING,
J. D. SKEER,
E. C. DUDLEY,
E. C. MANN,
H. T. BYFORD,
J. N. HYDE,
H. H. HALL,
W. W. GRANT,
J. W. NELSON.
M. MANNHEIMER,
L. L. MCARTHUR,
J. S. KNOX,
H. D. SCHMIDT,
C. W. EARLE,
A. B. STRONG,
F. E. WAXHAM,
G. A. STAPLES,

SUBSCRIPTION PRICE, $3.00 PER YEAR.

Whole Number, 313. JANUARY, 1886. Vol. LII., Number 1.

INDEX MEDICUS

LIBRARY INDEXED S.G.O.

### THE CHICAGO MEDICAL JOURNAL AND EXAMINER

EDITORS:

S. J. JONES, M.D., LL.D.

W. W. JAGGARD, A.M., M.D. HAROLD N. MOYER, M.D.

COLLABORATORS.

CHICAGO:

PROFESSOR J. A. ALLEN, M. D., LL. D.
PROFESSOR E. ANDREWS, M. D., LL. D.
PROFESSOR W. H. BYFORD, A. M., M. D.
PROFESSOR O. C. DEWOLF, A. M., M. D.
PROFESSOR E. C. DUDLEY, A. B., M. D.
PROFESSOR J. H. ETHERIDGE, A. M., M. D.
PROFESSOR C. FENGER, M. D.
PROFESSOR J. N. HYDE, A. M., M. D.
PROFESSOR E. F. INGALS, A. M., M.D.
PROFESSOR H. A. JOHNSON, M. D., LL. D.
CHARLES GILMAN SMITH, A. M., M.D,
J. F. TODD, M. D.

MILWAUKEE, WISCONSIN:
PROFESSOR N. SENN, M. D.

WASHINGTON, D. C:
S. O. RICHEY, M. D.

UNITED STATES ARMY:
SURGEON, D. L. HUNTINGTON, M. D.

UNITED STATES NAVY:
MEDICAL DIRECTOR, J. M. BROWNE, M. D
MEDICAL DIRECTOR, A L. GIHON, A. M., M. D

CHICAGO:

